# Impact of Nutritional Minerals Biomarkers on Cognitive Performance Among Bangladeshi Rural Adolescents—A Pilot Study

**DOI:** 10.3390/nu16223865

**Published:** 2024-11-13

**Authors:** Berna Rahi, Fahmida Rashid, Rasheda Sultana, Julia Benoit, Faruque Parvez, Khalid Khan

**Affiliations:** 1 Department of Human Sciences, College of Health Sciences, Sam Houston State University, Huntsville, TX 77341, USA; 2 Department of Public Health, College of Health Sciences, Sam Houston State University, Huntsville, TX 77341, USA; fxr040@shsu.edu (F.R.); mxs218@shsu.edu (R.S.); kxk051@shsu.edu (K.K.); 3 Texas Institute for Measurement, Evaluation, and Statistics, University of Houston, Houston, TX 77004, USA; julia.benoit@times.uh.edu; 4 Department of Environmental Health, Mailman School of Public Health, Columbia University, 722W, 168th St., New York, NY 10032, USA; mp844@cumc.columbia.edu

**Keywords:** nutritional minerals, neurocognitive function, adolescent, iron, zinc, selenium, cognitive performance, BARS

## Abstract

**Background:** Nutritional metals (NM) are essential for neurodevelopment and cognitive performance during growth. Nevertheless, epidemiological evidence regarding the associations between NM and brain function remains understudied, particularly among adolescents. Therefore, the objective of this pilot study was to examine the effects of NM biomarkers such as iron (Fe), selenium (Se), zinc (Zn), magnesium (Mg), and copper (Cu) on neurobehavioral functions among a group of rural Bangladeshi adolescents. **Methodology:** We conducted a cross-sectional study involving 105 adolescents aged 13–17 from Araihazar, Bangladesh. Cognitive function was assessed using the computer-based Behavioral Assessment and Research System (BARS), focusing attention, memory, and executive function, and blood NM levels (Fe, Se, Zn, Mg, and Cu) were measured. Associations between individual minerals, NM composite scores, and cognition were analyzed using multiple linear regressions. **Results:** This study included 47 boys and 58 girls with an average age of 15 years. Fe levels were correlated with Continuous Performance Test (CPT) latency (r = −0.42, *p* < 0.05) and Se levels correlated with Match-to-Sample (MTS) correct count (r = 0.32, *p* < 0.01). Linear regressions showed that Se was associated with MTS correct count (b = 0.02, 95%CI: 0.01, −0.04), reflecting visual memory, and Fe was associated with CPT latency (b = −0.68, 95%CI: −1.11, −0.26), reflecting improved attention. The same BARS measures were also significantly associated with the 3-NM composite score. **Conclusions:** Our findings suggest that NM, particularly Fe, Se, and NM mixtures, could play a crucial role in brain development and neurocognitive function during adolescence. Further studies will help design national public health policies and strategies to address and mitigate brain health deficiencies among adolescents.

## 1. Introduction

Nutrition plays a pivotal role in brain development and particularly in the first 1000 days of life [1,2,3]. Adequate nutrient intake from blood can ensure a healthy brain by impacting the levels of neurotransmitters and neuronal firing along with alteration of the structure of the membrane [4]. Among nutritional minerals (NM), the roles and mechanisms of iron (Fe), selenium (Se), zinc (Zn), magnesium (Mg), and copper (Cu) in maintaining brain functions are explained through animal and human studies. Fe ensures normal neurocognitive functions and induces proper development of brain cells producing myelin [5,6]. Zn is shown to be associated with attention, learning, memory, and neuropsychological behavior [7,8]. The positive impact of Mg on the proper functioning of nervous systems, including memory and attention, is evident [9], whereas Cu maintains the health of the nerves and blood vessels connected to the brain [10]. Se is an antioxidant that is preferentially retained in the brain [11,12], and its deficiency and that of its derivatives, the selenoproteins, may lead to multiple brain disorders in adults [13].

In early life stages, positive impacts of NM on the mental and cognitive growth of children have been reported [1,14,15,16]. Research has found that Fe deficiency during pregnancy could impair cognitive and behavioral development in their children [5,6]. The essential roles of Fe for myelination, synaptogenesis, and dendritic growth, which are key for brain development in early life, are also reported [17]. Furthermore, it has been suggested that eradicating the deficiencies of Fe and Zn could improve children’s IQ [14,18]. Zn is another important mineral for cognitive function, as its deficiency during pregnancy and early childhood is correlated with reduced attention, motor function, and learning outcomes [19]. For instance, Zn supplements among pregnant women and infants may lead to improved neuropsychological performance and better learning capabilities in childhood [14,20]. Similarly, Mg intake during pregnancy may produce beneficial effects on developing brains as it helps in maintaining healthy synaptic transmission and neural plasticity in early childhood [21,22,23,24,25,26]. Cu is also important for the developing fetus and infants because of its role in the synthesis of neurotransmitters and formation of myelin sheaths around neurons [27,28]. Cu deficiency has been shown to reduce IQ and delay mental and motor development in children [29]. Lastly, Se acts as a shield against harmful effects as an antioxidant [30]. Some research found that Se supplementation during pregnancy significantly improves cognitive outcomes in children by limiting oxidative stress and promoting neuronal health [31]. Although the majority of the studies examining NM and cognitive function have been performed in pregnant women and young children, whether and how these positive impacts sustain later in late childhood is not yet fully understood.

Adolescence is an important neurodevelopmental period in childhood, during which brain regions linked to executive function develop and mature [32]. In addition, during adolescence, growth and development are transformative and have profound consequences on an individual’s health in later life. Furthermore, the current generation of adolescents is growing up at a time of unprecedented change in food environments, whereby nutritional problems of micronutrient deficiency and food insecurity persist, and overweight and obesity are expanding [33]. Consequently, results of previously published studies in children and adults cannot be extrapolated to adolescents and will not provide an adequate insight into the development that rapidly emerges during adolescence [32]. Therefore, the interplay between NM status and neurocognitive outcomes in adolescents remains an essential area to explore, with specific emphasis on low-income communities where NM deficiencies are highly prevalent.

In Bangladesh, a Low- and Middle-Income Country (LMIC), a high proportion of children enter adolescence being underweight/stunted, and widespread micronutrient deficiencies have been captured among adolescents [34,35]. A recent study among 2463 Bangladeshi adolescents (51.2% girls) observed that more than half of the participants (53.9%) were food insecure and that they had low dietary diversity [36]. Therefore, the link between NM and neurocognitive function among Bangladeshi adolescents is of increased concern and requires further investigation. Nevertheless, to our knowledge, very few studies have examined these associations in this age group, while the majority of the studies focused on women of reproductive age, children, and older adults [37,38]. This creates a critical knowledge gap as the brain undergoes maturation during middle to late adolescence [39].

Therefore, this pilot study examines the associations of different NM in blood with the neurocognitive performance of Bangladeshi adolescents. In particular, we aim to determine if blood levels of Zn, Cu, Se, Fe, and Mg are associated with different measures of neurocognitive performance in this sample.

## 2. Materials and Methods

### 2.1. Study Area and Participants

We conducted a pilot study using a subset of adolescents who are participants of the ongoing Brain Health study funded by the National Institute of Health (NIH) (R01ES032149). One hundred and nine adolescents, aged 13 to 17 out of 300 participants, who provided blood samples for metal exposure assessment for the parent study were randomly identified and later included in our pilot study for further investigation. Since the primary aim and design of the study are exploratory, power analysis and sample size calculations were not performed.

Both the parent study and embedded pilot study were approved by the SHSU Institutional Review Board (IRB) (IRB-2021-180, approval date: 15 June 2022) and the Bangladesh Medical Research Council IRB. Parental-signed informed consent and child assent were obtained once the mother agreed to allow her adolescent child to participate in the study.

### 2.2. Procedure

The current pilot study utilized a subset of adolescent participants of the parent cross-sectional study taking place in Araihazar, Bangladesh. Participants with their mothers visited the Health Effects of Arsenic Longitudinal Study (HEALS) clinic, the field health care facility used for the parent study, for completing several activities. First, a whole blood sample of 5 mL was collected from each participant for metal analysis at the Columbia University Trace Metal Core laboratory (CU-TMC). Second, neurocognitive assessment using the computer-based Behavioral Assessment and Research System (BARS) was conducted in an enclosed private room facilitated by a trained tester. Third, measurements of height, weight, and age of the participants were collected. All of these sample and data collection activities were conducted under the supervision of a field research officer trained by the investigators of the study. The study sample collection, questionnaire survey, and neurocognitive assessments took place between 15 November 2022 and 22 June 2023.

### 2.3. Nutritional Minerals Assessment

A whole blood sample (12 mL) was collected in two 10 mL K2EDTA metal free tubes from each participant (6 mL in each tube) for the assessment of metal exposure and several other biomarkers (not relevant to the present study). Samples were immediately stored at −20 °C until shipped on dry ice to the CU-TMC laboratory in the United States. Assessments of Cu, Zn, and Se were conducted using a Perkin-Elmer NexION 350S equipped with an Elemental Scientific autosampler 4DX. Inductively Coupled Plasma Mass Spectrometry (ICP-MS)—with dynamic reaction cell (ICP-MS-DRC) methods for metals in whole blood were developed according to published procedures [40,41]. Additional analyses for Mg and Fe were performed for a subsample (n = 39). Whole blood samples were stored at negative 20 °C until shipped on dry ice to the CU-TMC laboratory in the USA, where nutritional minerals were measured by Inductively Coupled Plasma Mass Spectrometry (ICP-MS). The nutritional minerals were then used as continuous variables in the statistical analysis for the present pilot study.

### 2.4. Neurocognitive Performance Assessment

Neurocognitive function was examined through a computerized test battery, the Behavioral Assessment and Research System tests (BARS), which has been validated to be used among the rural Bangladeshi population [42]. The test battery included five tests to measure neurocognitive performance across various domains, such as Simple Reaction Time (SRT) for response speed, Match to Sample (MTS) for visual memory, Continuous Performance Test (CPT) for sustained visual attention, Symbol Digit Test (SDT) for information processing speed, and the Digit Span Test (DST) for assessing both attention and memory (Table 1). Prior computer knowledge was not required for completing the BARS. Nevertheless, the participants needed to know how to count from 1 to 9, as the response unit used in all tests is based on nine buttons labeled with one of these nine single-digit numbers. Participants were instructed by a trained tester to use one or more of the nine keys while performing the tests following a structured instruction manual in the local language (i.e., Bangla). In general, responses were based on two metrics: either time to respond (SRT latency, MTS latency, CPT latency, and SDT latency) or number of correct answers (MTS count, SDT errors, and DST forward and backward). Time to respond measures have milliseconds as a unit, and lower latencies indicated better neurocognitive performance. In contrast, a higher correct count indicated better performance.

Each test had two built-in components. First, a practice test (demo version) was presented to the participant to get familiarized with the test. Second, instructions (i.e., short sentences) required to perform the tests were translated from English to Bangla (local language) by the testers during the demo tests so that the participant could get clear step-by-step directions for the tests. Once the tests were completed, the results were calculated, aggregated, and stored automatically in computers using the BARS result mover (a component of the BARS Release 2.9 software). All BARS data were transferred by the HEALS informatics team in Bangladesh to the SHSU investigators in secured password-protected files for analyses. All BARS data were deidentified and contained only the identification number of the participants. All BARS variables were used as continuous variables during the statistical analysis. A structured questionnaire was used to collect sociodemographic information from the participants, including age and sex of the participants. Height and weight were measured before BARS data collection, and BMI categories were computed by plotting the BMI against the age of the adolescents on the CDC sex-specific BMI-for age growth charts. Four categories were created: underweight (<5th percentile), healthy weight (between the 5th and the 85th percentile), overweight (between the 85th and the 95th percentile), and obese (≥95th percentile).

### 2.5. Statistical Analysis

Descriptive analyses were performed to examine the characteristics of the whole sample and between boys and girls. Independent *t*-test and Chi-square tests were used for continuous and categorical variables, respectively. Pearson’s correlations were computed to determine the correlations between the levels of nutritional minerals and the BARS measures. In addition, to assess the combined effects of these minerals, we created a score based on the median level of each mineral. Levels were dichotomized and were given a score of 0 when level was below the median and a score of 1 if intake is above the median. We created this score for the whole sample, and it included Zn, Cu, and Se, and it ranged from 0 to 3, with 0 indicating that all three minerals had a level below the median while a score of 3 indicating all levels were above the median. We also calculated the same score for the subsample that included data for Fe and Mg (n = 37). Hence, this score included 5 minerals and ranged from 0 to 5. Based on the identified significant Pearson’s correlations, we performed multiple linear regressions to test the individual associations between different nutritional minerals, their combined effects, and cognitive performance among adolescents. Two models were tested. The first was a simple linear regression (crude model), and the second was adjusted for age, sex, and BMI categories. The normality of the data were analyzed by (1) visually assessing the distribution of the variables of interest by the frequency distribution, the P-P plot, and the Q-Q plot; (2) carrying out normality tests (Kolmogorov–Smirnov and Shapiro–Wilk) and checking the skewness and kurtosis of the distribution; (3) assessing the normality of the residuals; and (4) plotting the residuals against the predicted values. All performed analyses for the whole sample (n = 105) are presented here in the manuscript. The subsample analysis (n = 37) is reported as Supplementary Materials. All statistical analyses were performed using SPSS (V28), and statistical significance was set at *p* < 0.05.

### 2.6. Results

The final sample size of the current study was 105 participants, with four participants identified as outliers and excluded from the final analyses. The sociodemographic, anthropometric, and nutritional data are presented in Table 2, indicating no significant differences between boys and girls with respect to sociodemographic characteristics and nutritional biomarkers. The majority of the sample (56.2%) had a healthy weight, and approximately one third were classified as underweight (36.2%). Regarding the 3-NM composite score, the majority of the adolescents scored 1 (41%) or 2 (38.1%) NM above the median, with an average score of 1.5. This distribution was similar between boys and girls. Lastly, when the five NM were considered in the composite score, we observed that more than half of the participants (51.3%) had scores of 3 and 4, while two participants only (5.3%) had the blood NM levels above the median for all 5 minerals (Table 2). Baseline characteristics for the subsample (n = 37) are presented in Supplementary Table S1.

The neurocognitive performance of the whole sample, stratified by gender, is presented in Table 3 and Supplementary Table S2 (n = 37). In general, girls showed a better performance than boys, with significant differences observed for the symbol digit test (SDT) latency (2582.84 vs. 2955.72, *p* = 0.00) as well as match to sample (MTS) latency (2653.26 vs. 2863.45, *p* = 0.02).

Pearson’s correlations between the nutritional minerals and the neurocognitive measures of BARS were presented in Table 4 and Supplementary Table S3. Two significant correlations were only observed. First, Se was significantly correlated with MTS correct count (r = 0.32, *p* < 0.01), indicating that higher blood Se levels were correlated with better visual memory. Second, a significantly negative correlation between blood Fe levels and continuous performance test (CPT) latency (r = −0.42, *p* < 0.05) was observed, indicating that higher Fe levels were associated with a shorter response time, hence better sustained attention among Bangladeshi adolescents. All other correlations were not significant.

Linear regression models were developed to determine the associations between NM and neurocognitive performance. The results of the significant or borderline associations are presented in Table 5. In general, several individual NM showed associations with BARS measures. For instance, we observed that both blood levels of Zn and Fe were significantly associated with CPT latency, whereas Se levels were associated with MTS count score (Table 5). We observed that higher blood Fe was significantly associated with a lower response time on CPT latency. After adjustment for age, sex, and BMI categories (model 2), we observed that for every unit increase in Fe levels, the response time was decreased by 0.68 units (95% CI: −1.107, −0.257) (Table 5). Furthermore, blood Se was also associated with a higher count of correct answers on the MTS test, indicating better cognitive performance in both models (adjusted b = 0.024, 95%CI: 0.01, 0.037) (Table 5).

When considering the combined levels of the NM on neurocognitive performance, we observed that the 3-NM score was associated with CPT latency, MTS latency, and MTS count score. In fact, each unit increase in the 3-NM score (having one mineral above the median) was associated with a decrease of 16.39 ms (b adjusted = −16.39, 95%CI: −32.27, −0.50) on the CPT latency in the adjusted model and 120.07 ms (b = −120.07, 95%CI: −229.41, −10.73) on the MTS latency score in the crude model. This significance was borderline after adjustment for age, sex, and BMI. In addition, the 3-NM score was also significantly associated with the MTS count score, indicating better visual memory among our participants. The results indicated that for each unit increase in the composite score, the number of correct answers in the MTS count score increased by 0.46 (adjusted b = 0.455, 95% CI: 0.0003, 0.906) (Supplementary Table S4).

Lastly, the associations between the BARS measures and the 5-NM composite score demonstrated borderline significance for CPT latency, MTS latency, and MTS count score (Supplementary Table S4). Overall, our results imply that these NM may have positive impacts on several domains of neurocogntive performance, such as visual memory and attention.

## 3. Discussion

Our study demonstrated significant associations of several key NM with neurocognitive outcomes in an adolescent sample from a low-income population. More specifically, we found beneficial effects of Se and Fe on enhanced attention and visual memory while analyzing CPT latency and MTS count data with respect to these two NM. Our findings also revealed potential effects of mineral mixtures (i.e., the 3-NM composite score) on visual and attention measures of neurocognitive performance.

These results contribute to the growing body of evidence suggesting the role of Se in brain development [43]. Our results are consistent with previous research among 572 Bangladeshi adolescents aged 14–16 years where blood Se levels showed a consistent positive association with cognition [44]. Skröder, Kippler et al. 2017, also observed that prenatal and childhood Se status is associated with a higher cognitive function score at 5 and 10 years of age among 1408 children included in a study nested in a population-based, randomized supplementation trial during pregnancy in rural Bangladesh [45].

The role of Fe in neurocognitive function in early childhood is crucial since its effect on brain development during the first two years of life is well established [2,3,4]. In fact, Fe is necessary for myelination, neurogenesis, and neuronal differentiation [46]. Several studies observed that higher Fe levels during the pre-natal and post-natal period and early childhood were associated with better cognitive performance, motor development, and psychomotor skills [47]. Our study showed that higher Fe levels were associated with better cognitive performance, including shorter response times and improved sustained attention during adolescence. These results are in line with the results of a systematic review and meta-analysis that included 9 studies with 1196 children and adolescents aged 5 to 19 years and observed that Fe had a positive impact on intelligence test scores while no significant effects were observed on attention and memory [48]. Similarly, another systematic review suggested that Fe status and anemia might be associated with academic performance in some contexts and that Fe supplementation during adolescence may improve school performance, attention, and concentration, while no evidence suggested Fe influences on intelligence or memory in adolescents [49]. Lastly, a longitudinal study of 922 participants aged 8–26 years observed that greater cognitive ability is increasingly associated with greater Fe concentration through late adolescence and young adulthood [46].

In our analyses, blood Zn demonstrated a borderline significant association with reduced CPT latency, indicating a potential positive impact on attention. Several prior studies involving similar age groups investigated this impact and observed significant effects. For instance, a cross-sectional study in 100 high school female students observed serum Zn positively correlated with various domains of neurocognitive function in young female adolescents [50]. Similarly, cognitive performance was also linked to supplementation with zinc rich foods among 180 Indian girls aged 12 years [51]. A systematic review and a meta-analysis on Zn intake/status or supplementation and cognitive function in adults and children showed mixed results on cognitive functioning in children, concluding the necessity of high-quality studies to further investigate these effects [52]. A more recent peer review examining the associations between Zn intake, through diet or supplement, Zn status (plasma/serum), and the different domains of cognitive functions in school-aged children also observed inconsistencies [53]. Findings of these previous studies, along with our findings of borderline associations, necessitate the design and implementation of large-scale epidemiological studies with a larger sample size. This is important because Zn is an essential trace element for neurotransmission, synaptic plasticity, and the regulation of brain signaling pathways [8,54].

An innovative aspect of our study was to investigate the synergistic role of the different NM by creating two composite scores: the first included the three NM (3-NM) assessed in the whole sample (n = 105) and the second included five NM (5-NM) assessed in a sub-sample (n = 37), which had blood biomarker data for all five NM. Our results indicated that the 3-NM score was significantly associated with three neurocognitive outcomes of attention and visual memory, which were more promising findings than what we observed for individual NM. Moreover, the 5-NM showed borderline significance with the same BARS measures as the 3-NM. These observations are providing hints of potential synergistic mechanisms of NM mixtures on neurocognitive performance. These results are consistent with another study assessing the relationship between mixtures of NM and cognitive functions among 2428 participants aged 6–16 years recruited from 60 schools in India. The authors concluded that ≥2 micronutrient deficiencies were associated with impairment in cognitive function, specific to attention and concentration, visual-spatial ability, and/or working memory [55].

Our study is among the very few investigations that have addressed the impact of NM on the adolescent brain. Working with this age group in particular has public health implications since adolescence is a time of phenomenal growth where every physiological system is transformed and must be studied [56]. Particularly, adolescence is a critical neurodevelopmental period for brain development and maturation, making this phase of growth a pivotal window to correct any deficits [56,57]. Nevertheless, despite ongoing issues like micronutrient deficiencies resulting from food insecurity, adolescent nutrition has been largely neglected in global policies [57]. Nutritional investments in the first 1000 days of life are very important as they can successfully reduce child mortality and early childhood stunting and wasting [58]. However, the impacts of micronutrient exposure in late childhood received less attention. Investigations on micronutrient–brain associations during adolescence will benefit predicting neurocognition during adulthood, leading to more meaningful brain mapping throughout the life course [33,57]. Lastly, adolescence is the time of transition from primary dependence on caregivers to increasingly diverse roles and responsibilities related to food acquisition, preparation, and consumption, presenting a unique opportunity to foster healthy eating [59]. Therefore, to foster healthy eating during adolescence, an in-depth understanding of the role of nutrition among adolescents is essential, leading to better-designed public health campaigns and policies tailored for adolescents.

An innovative aspect of our pilot study was to examine the combined effects of blood NM levels by creating composite scores, specifically to correlate the synergistic effects of NM and neurocognitive function. Although the methodology used to create these composite scores is not yet validated, and our results need to be interpreted with caution, this approach showed promising results, paving the way for future research with a more refined statistical approach. Lastly, we employed a culturally adapted computer test battery (BARS), which has already been piloted in rural Bangladesh in children [42]. BARS has the ability to precisely measure multiple cognitive functions such as attention, memory, learning motivation, complex function, response speed, and coordination in a time-efficient manner and with limited or no involvement of the test administrator.

Despite these strengths, this study has limitations. First, this is an exploratory study that has methodological limitations. Its cross-sectional design cannot allow for the establishment of temporal associations, and the relatively small sample size, especially for Fe and Mg, makes the statistical power to explore the associations between NM and neurocognitive performance limited. In addition, we are aware that the relationships of NM and cognition may be confounded by several other relevant confounding factors, including other nutritional factors (deficiencies, food insecurity, interactions with other NMs), socio-economic status, or toxic metal exposures. We were not able to adjust for these confounders because of the exploratory nature as well as budget and time constraints in our study. Second, although considered a gold standard method, blood level measurements have limitations that need to be addressed as they can be associated with the physiological state of the participants. For instance, Zn in serum can be associated with the sex, age, as well as the time of blood draw (morning vs evening) [60]. They also fluctuate in response to other factors, including infections, changes in steroid hormones, and muscle catabolism during weight loss or illness [61]. Third, the lack of dietary information in this exploratory study prevented us from correlating blood levels of NM with dietary intake, which could have helped examine the role of the adolescents’ dietary habits in determining these associations. Lastly, limitations associated with conducting the study in an LMIC warrant careful consideration. These countries often experience elevated levels of toxic metal exposure [62], which can significantly interfere with the impact of NMs on brain development. Unfortunately, this study did not account for these potential influences on neurocognitive performance. Despite these limitations, our preliminary findings may lay the foundation for a larger study with adequate statistical power and advanced statistical methods to account for the synergistic effects of different NMs on cognitive performance among adolescents.

## 4. Conclusions

Our preliminary findings suggest that NM, particularly Fe, Se, and NM mixtures, could play a crucial role in brain development and neurocognitive function during adolescence, a critically important age group that is yet to be fully investigated with respect to NM. Large-scale epidemiological investigations with larger sample sizes and more robust study designs would be necessary to confirm these preliminary findings and to fully understand the combined effects of NM on adolescent brain health. Eventually, studies in this field will help design national public health policies and strategies to address and mitigate brain health deficiencies not only for adolescence but also for the entire life span.

## Figures and Tables

**Table 1:** BARS measures and their cognition domain.

BARS Measures	Domain of Cognition Measured
Match to Sample	Visual Memory
Digit Span	Short-term Memory, Attention
Continuous Performance	Sustained Attention
Finger Tapping	Motor Coordination
Simple Reaction Time	Response Speed
Symbol Digit	Information Processing Speed

**Table 2:** Baseline sociodemographic data for the whole sample of adolescents (n = 105) and stratified by gender.

Variable	Whole Sample n = 105	Boys n = 47	Girls n = 58	*p*-Value
Age	15.05 ± 1.26	15.02 ± 1.29	15.07 ± 1.25	0.85
Girls, n (%)	58 (55.2)			
BMI categories, n (%)				0.50
Underweight	38 (36.2)	18 (38.3)	20 (34.5)	
Healthy weight	59 (56.2)	27 (57.4)	32 (55.2)	
Overweight/obese	8 (7.6)	2 (4.3)	6 (10.3)	
Nutritional biomarkers				
Zn (µg/L)	5231.17 ± 1033.40	5181.64 ± 966.24	5271.31 ± 1091.50	0.66
Cu (µg/L)	794.56 ± 100.11	776.20 ± 95.51	809.44 ± 102.08	0.09
Se (µg/L)	129.43 ± 26.15	129.90 ± 22.95	129.05 ± 28.67	0.87
Mg (mg/L)	29.69 ± 3.43	30.88 ± 3.92	28.96 ± 2.95	0.10
Fe (mg/L)	370.12 ± 55.65	392.95 ± 62.74	356.22 ± 46.99	0.07
Composite Minerals Scores				
3-NM score (continuous)	1.5 ± 0.8	1.5 ± 0.9	1.5 ± 0.78	0.97
3-NM Score categories, n (%)				0.59
0	10 (9.5%)	5 (10.6%)	5 (8.6%)	
1	43 (41%)	20 (42.6%)	23 (39.7%)	
2	40 (38.1%)	15 (31.95)	25 (43.1%)	
3	12 (11.4%)	7 (14.9%)	5 (8.6%)	
5-NM score (continuous)	2.6 ± 1.5	2.4 ± 1.7	2.6 ± 1.3	0.66
5-NM score (categories, n (%)				0.42
0	4 (10.8%)	3 (21.4%)	1 (4.3%)	
1	6 (16.2%)	1(7.1%)	5 (21.7%)	
2	6 (16.2%)	3 (21.4%)	3 (13.0%)	
3	9 (24.3%)	2 (14.3%)	7 (30.4%)	
4	10 (27.0%)	4 (28.6%)	6 (26.1%)	
5	2 (5.45%)	1 (7.1%)	1 (4.3%)	

Data are presented as mean ± SD unless specified otherwise. Differences between boys and girls were tested by *t*-tests or Chi-Square tests depending on the type of variable. The statistical level was set at *p* < 0.05. n = 105 except for Mg and Fe, where n = 37, with n = 23 as girls.

**Table 3:** Neurocognitive performance measures for the whole sample and stratified by gender (n = 105).

Variable	Whole Sample n = 105	Boys n = 47	Girls n = 58	*p*-Value
SRT Latency (ms)	328.4 ± 40.1	330.3 ± 38.9	326.8 ± 41.2	0.66
**SDT Latency (ms)**	**2749.75 ± 616.20**	**2955.72 ± 662.33**	**2582.84 ± 524.83**	**<0.05**
DST forward (count)	4.79 ± 1.07	4.77 ± 1.1	4.8 ± 1.0	0.83
DST reverse (count)	3.36 ± 1.45	3.36 ± 1.4	3.36 ± 1.5	0.99
CPT Latency (ms)	327.12 ± 66.99	318.5 ± 76.4	334.1 ± 58.0	0.25
MTS count score	17.06 ± 1.88	17.0 ± 1.9	17.14 ± 1.8	0.63
**MTS Latency (ms)**	**2747.34 ± 470.26**	**2863.45 ± 488.12**	**2653.26 ± 437.13**	**<0.05**

Data are presented as mean ± SD. Differences between boys and girls were tested by independent *t*-tests. Bold values indicate significant differences (*p* < 0.05).

**Table 4:** Pearson’s Correlations between nutritional minerals and neurocognitive function as measured by BARS.

Variable	Zn (µg/L) (n = 105)	Cu (µg/L) (n = 105)	Se (µg/L) (n = 105)	Mg (mg/L) (n = 37)	Fe (mg/L) (n = 37)
SRT Latency (ms)	−0.07	0.17	−0.10	−0.17	−0.27
SDT Latency (ms)	−0.11	0.12	−0.04	−0.03	0.05
DST forward (count)	−0.17	−0.02	0.04	0.16	0.11
DST reverse (count)	0.05	−0.03	−0.1	0.11	0.01
CPT Latency (ms)	−0.17	0.04	−0.07	0.21	−0.42 *
MTS count score	−0.09	−0.05	0.32 **	0.25	0.06
MTS Latency (ms)	−0.19	−0.1	0.02	−0.04	−0.18

* *p* < 0.05. ** *p* < 0.01.

**Table 5:** Linear regression analyses results between the nutritional minerals and different measures of BARS among Bangladeshi Adolescents.

	CPT Latency (ms)		MTS Latency (ms)		SRT Latency (ms)		SDT Latency (ms)		DST Forward		DST Reverse		MTS Count	
	Crude Model b (95% CI)	Adjusted Model b (95% CI)	Crude Model b (95% CI)	Adjusted Model b (95% CI)	Crude Model b (95% CI)	Adjusted Model b (95% CI)	Crude Model b (95% CI)	Adjusted Model b (95% CI)	Crude Model b (95% CI)	Adjusted Model b (95% CI)	Crude Model b (95% CI)	Adjusted Model b (95% CI)	Crude Model b (95% CI)	Adjusted Model b (95% CI)
Zn (µg/L)	−0.01 (−0.02, 0.00)	−0.02 * (−0.03, 0.00)	−0.09 * (−0.17, 0.00)	−0.08 (−0.17, 0.01)	−0.00 (−0.01, 0.01)	−0.01 (−0.01, 0.01)	−0.067 (−0.18, 0.05)	−0.02 (−0.13, 0.09)	0.00 (0.00, 0.00)	0.00 * (0.00, 0.00)	0.00 (0.00, 0.00)	0.00 (0.00, 0.00)	0.00 (−0.00, 0.00)	0.00 (−0.00, 0.00)
Cu (µg/L)	0.02 (−0.11, 0.16)	0.03 (−0.11, 0.16)	−0.46 (−1.37, 0.46)	−0.15 (−1.07, 0.77)	0.06 (−0.01, 0.15)	0.07 (−0.01, 0.15)	0.76 (−0.43, 1.96)	0.90 (−0.25, 2.05)	0.00 (−0.00, 0.00)	0.00 (−0.00, 0.00)	0.00 (−0.00, 0.00)	0.00 (−0.00, 0.00)	−0.00 (−0.01, 0.00)	−0.00 (−0.01, 0.00)
Se (µg/L)	−0.17 (−0.67, 0.33)	−0.18 (−0.68, 0.33)	0.43 (−3.09, 3.94)	0.59 (−2.82, 3.99)	−0.15 (−0.45, 0.14)	−0.16 (−0.46, 0.14)	−0.96 (−5.56, 3.64)	−0.86 (−5.17, 3.45)	0.00 (−0.01, 0.01)	0.00 (−0.01, 0.01)	−0.01 (−0.02, 0.01)	−0.01 (−0.02, 0.01)	0.02 *** (0.01, 0.04)	0.02 *** (0.01, 0.04)
Composite Score (3 Metals)	−15.71 * (−31.34, −0.08)	−16.39 * (−32.27, −0.50)	−120.07 * (−229.41, −10.73)	−100.41 (−208.70, 7.88)	−1.80 (−11.33, 7.72)	−2.89 (−12.64, 6.86)	−97.46 (−242.76, 47.83)	−92.62 (−230.99, 45.75)	0.10 (−0.15, 0.36)	0.10 (−0.17, 0.36)	0.01 (−0.34, 0.35)	−0.01 (−0.36, 0.34)	0.40 (−0.04, 0.84)	0.46 * (0.003, 0.91)

Model 1: crude model; Model 2: adjusted for age, sex, and BMI categories. * *p* < 0.05. *** *p* < 0.001.

## Data Availability

The original contributions presented in the study are included in the article/Supplementary Material, further inquiries can be directed to the corresponding author.

## Supplementary Materials

The following supporting information can be downloaded at: https://www.mdpi.com/article/10.3390/nu16223865/s1, Table S1: Baseline sociodemographic data for the whole sample of adolescents (n = 37) and stratified by gender. Table S2: Neurocognitive performance measures for the whole sample and stratified by gender (n = 37). Table S3: Pearson’s Correlations between nutritional minerals and neurocognitive function as measured by BARS for n = 37. Table S4: Linear regression analyses results between the nutritional minerals and different measures of BARS among Bangladeshi Adolescents (n = 37).
