# Supplementary material for: Impact of Nutritional Minerals Biomarkers on Cognitive Performance Among Bangladeshi Rural Adolescents—A Pilot Study
Source: Nutrients. 2024 Nov 13;16(22):3865. doi: 10.3390/nu16223865 (PMC11597576; doi:10.3390/nu16223865)
Supplement: Supplementary file 1 [file nutrients-16-03865-s001.zip › nutrients-3292197-supplementary.pdf]

Supplemental Tables:

Table S1: Baseline sociodemographic data for the whole sample of adolescents (n=37) and stratified by gender.

Variable	Whole sample n=37	Boys	Girls	p
Age	15.4 ± 1.3	15.4 ± 1.4	15.4 ± 1.3	0.86
Girls, (n%)	23 (62.2%)			
BMI categories, n(%)				
Underweight	6 (16.2%)	3 (21.4%)	3 (13%)	0.78
Healthy weight	26 (16.2%)	9 (64.3%)	17 (73.9%)	
Overweight/obese	5 (13.5%)	2 (14.3%)	3 (13.3%)	
Nutritional biomarkers				
Zn (µg/L)	5832.7 ± 988.4	5574 ± 1101.3	5989 ± 902.1	0.25
Cu (µg/L)	804.2 ± 99.5	766.2 ± 102.4	827.3 ± 92.4	0.79
Se (µg/L)	110.0 ± 18.5	110.4 ± 20.1	109.7 ± 18.0	0.91
Mg (mg/L)	29.7 ± 3.4	30.9 ± 3.9	29.0 ± 3.0	0.13
Fe (mg/L)	370.1 ± 55.6	392.9 ± 62.7	356.2 ± 47.0	0.07

Data is presented as mean ± SD unless specified otherwise. Differences between boys and girls were tested by t-tests or Chi-Square tests depending on the type of variable. The statistical level set at $p<0.05$.

Table S2: Neurocognitive performance measures for the whole sample and stratified by gender (n=37)

Variable	Whole sample Mean ± SD n=37	Boys	Girls	p
SRT Latency (ms)	335.0 ± 43.5	341.4 ± 45.7	331.1 ± 42.6	0.50
SDT Latency (ms)	2767.2 ± 633.1	3097.4 ± 547.7	2566.3 ± 605.9	0.01**
SDT errors (count)	2.5 ± 3.4	2.5 ± 2.2	2.5 ± 4	0.98
DST forward (count)	4.6 ± 1.1	4.9 ± 1.2	4.5 ± 1.1	0.26
DST reverse (count)	3.6 ± 1.7	4.1 ± 1.6	3.3 ± 1.8	0.14
CPT Latency (ms)	340.3 ± 71.7	350.25 ± 87.6	334.2 ± 61.4	0.55
MTS count score	16.3 ± 1.8	16.2 ± 1.9	16.3 ± 1.8	0.88
MTS Latency (ms)	2633 ± 403.8	2841.5 ± 434.5	2507 ± 333.3	0.02*

Data is presented as mean ± SD. Differences between boys and girls were tested by independent t-tests.

*$p<0.05$

**$p<0.01$

Table S3: Pearson's Correlations between nutritional minerals and neurocognitive function as measured by BARS for n=37.

Variable	Zn (µg/L)	Cu (µg/L)	Se (µg/L)	Mg (mg/L)	Fe (mg/L)
SRT Latency (ms)	-0.14	0.13	-0.13	-0.2	-0.27
**SDT Latency (ms)**	-0.29	-0.15	-0.21	-0.33	0.05
DST forward (count)	0.07	0.03	0.02	0.16	0.11
DST reverse (count)	0.14	0.15	-0.02	0.11	0.007
CPT Latency (ms)	-0.39*	0.26	-0.21	-0.21	-0.42**
MTS count score	0.22	0.19	0.21	0.25	0.06
**MTS Latency (ms)**	-0.33**	-0.27	-0.19	-0.04	-0.18

*p<0.05

**p<0.01

Table S4: Linear regression analyses results between the nutritional minerals and different measures of BARS among Bangladeshi Adolescents (n=37)

	CPT Latency (ms)		MTS latency (ms)		SRT latency (ms)		SDT latency (ms)		DST forward		DST reverse		MTS count	
	Crude Model b (95% CI)	Adjusted Model b (95% CI)	Crude Model b (95% CI)	Adjusted Model b (95% CI)	Crude Model b (95% CI)	Adjusted Model b (95% CI)	Crude Model b (95% CI)	Adjusted Model b (95% CI)	Crude Model b (95% CI)	Adjusted Model b (95% CI)	Crude Model b (95% CI)	Adjusted Model b (95% CI)	Crude Model b (95% CI)	Adjusted Model b (95% CI)
Zn(µg/L)	-0.03** (-0.05, -0.01)	-0.03** (-0.06, -0.01)	-0.14* (-0.27, -0.00)	-0.12 (-0.25, 0.02)	-0.01 (-0.02, 0.01)	- 0.01 (-0.02, 0.01)	- 0.18 (-0.39, 0.03)	- 0.11 (-0.31, 0.10)	7.81E-5 (0.00, 0.00)	0.00 (0.00, 0.00)	0.00 (0.00, 0.00)	0.00 (0.00, 0.00)	0.00 (0.00, 0.00)	0.00 (0.00, 0.00)
Cu (µg/L)	0.19 (-0.05, 0.43)	0.24* (0.00, 0.49)	-1.10 (-2.44, 0.24)	-0.63 (-1.98, 0.72)	0.06 (-0.09, 0.20)	0.08 (-0.08, 0.23)	-0.94 (-3.10, 1.22)	-0.20 (-2.22, 1.81)	0.00 (- 0.00, 0.00)	0.00 (-0.00, 0.01)	0.00 (-0.00, 0.09)	0.00 (-0.00, 0.01)	0.00 (-0.00, 0.01)	0.00 (-0.00, 0.01)
Se (µg/L)	- 0.80 (-2.10, 0.50)	-1.14 (-2.50, 0.22)	-4.20 (-11.53, 3.14)	-5.29 (-12.59, 2.01)	- 0.31 (-1.11, 0.49)	-0.62 (-1.46, 0.23)	-7.33 (-18.78, 4.12)	-5.64 (-16.56, 5.27)	0.00 (-0.02, 0.02)	0.00 (-0.02, 0.02)	-0.00 (-0.03, 0.03)	-0.01 (-0.04, 0.03)	0.02 (-0.01, 0.05)	0.03 (-0.00, 0.07)
Mg (mg/L)	-4.43 (-11.43, 2.58)	-5.02 (-12.25, 2.20)	-4.21 (-44.56, 36.13)	-16.64 (-55.67, 22.40)	-2.49 (-6.78, 1.77)	-3.15 (-7.57, 1.28)	-6.13 (-69.40, 57.14)	-33.70 (-90.59, 23.20)	0.05 (-0.06, 0.16)	0.04 (-0.08, 0.16)	0.06 (-0.11, 0.23)	0.04 (-0.14, 0.22)	0.13 (-0.04, 0.31)	0.16 (-0.02, 0.34)
Fe (mg/L)	-0.54** (-0.94, -0.14)	-0.68* (-1.11, -0.26)	-1.32 (-3.77, 1.12)	-2.52* (-4.96, -0.09)	-0.21 (-0.47, 0.05)	-0.34** (-0.61, -0.06)	0.60 (-3.30, 4.50)	-1.29 (-5.10, 2.51)	0.00 (-0.01, 0.01)	0.00 (-0.01, 0.01)	0.00 (-0.01, 0.01)	-0.00 (-0.01, 0.01)	0.00 (-0.01, 0.01)	0.01 (-0.01, 0.02)
Composite Score (5 Metals)	-14.40[#] (-30.46, 1.66)	-17.27* (-35.14, 0.60)	-87.01* (-176.87, 2.86)	-88.69[#] (-183.80, 6.42)	-4.15 (-14.24, 5.94)	-7.97 (-19.21, 3.27)	-118.93 (-261.69, 23.83)	-121.23 (-262.42, 19.95)	0.03 (-0.24, 0.30)	0.08 (-0.22, 0.39)	-0.12 (-0.52, 0.28)	-0.07 (-0.52, 0.38)	0.03 (-0.39, 0.45)	0.22 (-0.27, 0.70)

Adjusted model include Age, Sex and BMI categories

*p<0.05

**p<0.01

***p<0.001

[#]p≤0.07
